# Stimulus-specific influence of gender on mental-rotation-related brain activity

**DOI:** 10.3758/s13415-025-01325-w

**Published:** 2025-07-01

**Authors:** Nadia M. Bersier, Raffaella I. Rumiati, Silvio Ionta

**Affiliations:** 1 SensoriMotorLab, Department of Ophthalmology-University of Lausanne, Jules Gonin Eye Hospital-Fondation Asile Des Aveugles, Avenue de France 15, 1004 Lausanne, Switzerland; 2 Area of Neuroscience, Scuola Internazionale Superiore Di Studi Avanzati, Trieste, Italy; 3 Department of Systems Medicine, University Rome ‘Tor Vergata’, Rome, Italy

**Keywords:** Gender differences, Mental rotation, FMRI, Bottom-up, Stimuli, Somatosensory processes

## Abstract

Mental rotation (MR) is a cognitive process during which individuals mentally simulate the rotation in space of an object’s image (stimulus). The traditional assertion that men outperform women in MR tasks may be influenced by methodological biases, such as treating gender as a secondary or post hoc variable, and relying solely on binary comparisons between two classes of MR stimuli. Furthermore, a comprehensive understanding of how nuanced the effects of the interaction between gender and stimulus type are on MR-related brain activity remains lacking. To fill these gaps, we recorded functional magnetic resonance imaging (fMRI) data while 57 participants (29 women, aged 18–35 years) performed MR of abstract objects, human bodies, and real objects. Whole-brain fMRI data analyses indicated that, with respect to women, men had larger activations in inferior frontal regions during MR of abstract objects, and in superior and medial frontal regions during MR of human bodies. Compared with men, in women we found larger activity in the superior parietal lobe during MR of human bodies with respect to abstract objects, and in the inferior occipital cortex in the MR of real objects versus human bodies. Finally, while in men we found a positive correlation between MR accuracy and brain activity in the precuneus, in women the correlation between MR accuracy and activity in motor and premotor areas was negative. These results indicate that brain activity during MR is modulated by the type of stimulus, differently for women and men.

## Introduction

Mental rotation (MR) is the cognitive ability to mentally perform spatial rotational transformations of images (stimuli) depicting objects, body parts, figures, etc. The characteristics of both the stimuli and the performers themselves can affect MR. While these influences have been traditionally linked to sharp, binary distinctions between two levels of a given factor (e.g., MR of one vs. another object), recent behavioral evidence is starting to reveal more nuanced effects due to interactions between factors. The present study investigates the neural bases of these refined interactions, with a particular focus on the relationship between stimulus type and participant gender.

Typical binary distinctions between the influence of different stimuli on MR include semantics (e.g., MR of one type of stimulus vs. another type; Dalecki et al., 2012, 2013; Jansen et al., 2020; Zeugin et al., 2020), viewpoint (e.g., MR of dorsum-view vs. palm-view hands; Bek et al., 2022; Schmid & Coppieters, 2012; Stone et al., 2019), visibility (e.g., MR of stimuli with high vs. low visibility; Giovaola et al., 2022; Rotach et al., 2024) and sensory modality (e.g., MR of visual vs. tactile stimuli; Caissie et al., 2018; Pamplona et al., 2022; Sveistrup et al., 2023). However, recent behavioral evidence suggests that the influence of stimuli’s features on MR is less sharp and more graded (Rajeb et al., 2024). This passage of emphasis from binary to nuanced modulations of MR is supported by brain imaging data. Typical binary comparisons of MR-related brain activations showed that MR of objects versus hands, respectively, is impaired by lesions in the right versus left hemisphere (Tomasino et al., 2003), and activates parieto-occipital versus sensorimotor regions (Aso et al., 2007; Kosslyn et al., 1998; Tomasino et al., 2004). In addition, different brain activations are associated with MR of abstract objects versus human bodies (Jansen et al., 2020), letters versus hands or scenes (Thomas et al., 2013), and tools versus hands (Vingerhoets et al., 2002). These and other studies suggest that previous sharp binary classifications may be in fact more gradual. For instance, MR of the same stimulus (hand) in different visual contexts (alone vs. attached to a body) activates different brain networks (Perruchoud et al., 2016), and MR of different stimuli does not necessarily activate different brain regions (Jordan et al., 2001).

Regarding the performer-related effects, gender is among the most typical factors appearing to play a central role in MR. Previous work reported that gender differences in MR emerge as early as 3 or 4 months after birth (Enge et al., 2023; Johnson & Moore, 2020; Quinn & Liben, 2008) and remain in adulthood (Doyle & Voyer, 2013; Halpern, 2013; Linn & Petersen, 1985; Long et al., 2021; Raabe et al., 2006; Shepard & Metzler, 1971; Voyer et al., 1995; Zapf et al., 2015). In addition, gender modulates the difficulty to perform MR of abstract objects (Campbell et al., 2018) and drives different brain activations associated with MR (Hugdahl et al., 2006; Jordan et al., 2002; Seurinck et al., 2004). However, gender differences can be in fact modulated by the type of MR stimulus (Alexander & Evardone, 2008; Doyle et al., 2016) and cognitive strategy used to perform MR (Bersier et al., 2024; Voyer et al., 2017). Such an interaction between stimulus type and gender is further supported by evidence that MR performance is better when the gender of the stimuli is congruent with the gender of the participant (Ruthsatz et al., 2019). Thus, gender differences in MR may be not as sharp as traditionally thought, because they can be abolished when MR (i) is performed with specific stimuli (Fisher et al., 2018; Jansen-Osmann & Heil, 2007; Rahe et al., 2018), (ii) occurs in specific settings (Parsons et al., 2004), and (iii) is controlled for other participant characteristics (Jansen et al., 2020; Moen et al., 2020; Tan et al., 2003).

In sum, previous behavioral and brain imaging investigations on the influence of stimuli and gender on MR-related brain activity and performance produced mixed results. This inconsistence may be due to methodological choices, such as using only two types of stimuli or the selection of gender as a post hoc factor. In addition, using only two types of MR stimuli risks to intrinsically limit the conclusions, because they would be based on only two extremes of what, in fact, could be a continuum between stimuli with progressively different levels of abstraction. Finally, with respect to primary factors, introducing gender as post hoc factor without predefined hypotheses and dedicated experimental designs may increase the risk of finding false positives.

A study specifically designed to tackle the role of gender and comprising more than only two types of stimuli would better address the interaction between stimuli- and gender-related effects on MR. Regarding the stimuli, MR of manipulable real objects may result in different behavioral performance and/or brain activity compared to less manipulable abstract objects. In addition, the implementation of bodily stimuli may allow to explore the influence of embodied cognition, a factor that could be overlooked if only objects would be used. For these reasons, in the present study we investigated whether the behavioral and neural effects of gender on MR may be sensitive to the type of stimulus (abstract objects, human bodies, and real objects). We predicted that (i) men would outperform women in the MR task in terms of accuracy and response times (RTs), (ii) MR-related brain activity would be different between men and women, and that (iii) the relationship between gender, MR performance, and brain activity would be further modulated by the type of stimuli. This approach aimed at advancing the field of MR by offering a more nuanced examination of how stimulus properties, further depending on gender, modulate the cognitive and neural processes involved in MR.

## Materials and methods

### Participants

A power analysis based on the fMRI data of a previous study with a similar experimental protocol (Bersier et al., 2024) indicated that a minimum of 49 participants would be required to detect a significant main effect of gender with a power of 0.8 and α = 0.05, assuming a medium effect size (Cohen’s d ≈ 0.5). To ensure statistical power even in case of data loss due to dropouts, MR performance at chance level, or excessive head motion, we recruited 60 participants. Data from three participants were excluded due to chance-level performance on the MR task. The definitive sample comprised 57 participants, including 29 women (mean age 24.17 years; standard deviations [SD] = 4.12 years; range 19–35 years) and 28 men (mean age 25.29 years; SD = 4.25 years; range 20–35 years). This sample consisted of university students with a differentiated range of academic backgrounds, including science, technology, engineering, mathematics, social sciences, humanities, life sciences, and psychology. Prior to the experiment, each participant signed a written informed consent, completed a magnetic resonance safety questionnaire, and declared their perceived gender identity. Participants were unaware of the research questions until the end of the experiment. Exclusion criteria were: age outside the pre-determined range (18–35 years); visual impairment without the possibility of correction; contraindication to fMRI (e.g., neurological/psychiatric disorder, claustrophobia, pregnancy, ferromagnetic implant); left-handedness as evaluated by a score below than the cutoff of 60 on the Edinburgh Handedness Inventory (Oldfield, 1971). The experimental procedures were approved by the local ethics committee and the experiment was conducted in accordance with the Declaration of Helsinki (2013).

### Stimuli

**Fig. 1:**
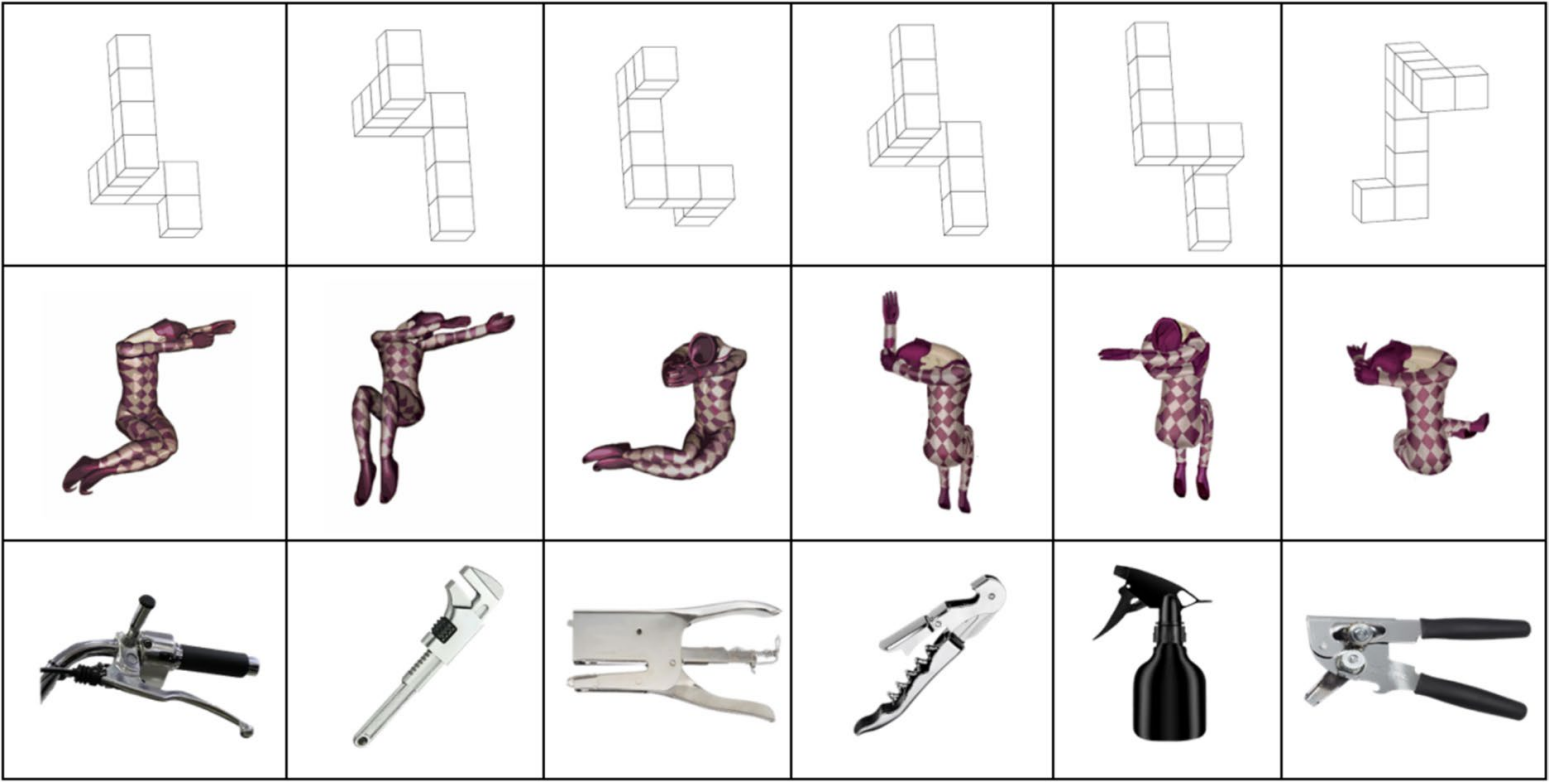
Experimental stimuli consisted in six abstract objects (top row), six human bodies (mid row), and six real objects (bottom row)

During fMRI data recording, participants performed MR of three types of stimuli: abstract objects, human bodies, and real objects (Fig. 1). Each class of stimuli comprised six items. The stimuli representing abstract objects resembled 3D cubic figures, such as those used by Shepard and Metzler (1971). The stimuli depicting human bodies were taken from Amorim et al. (2006) and represented a human body in six different postures. The six stimuli classified as real objects represented daily-use objects that could be grasped with one hand (bike break, wrench, stapler, corkscrew, spray bottle, can opener). All stimuli were digitally cleared to assure absence of writings and same overall size.

### Procedure

**Fig. 2:**
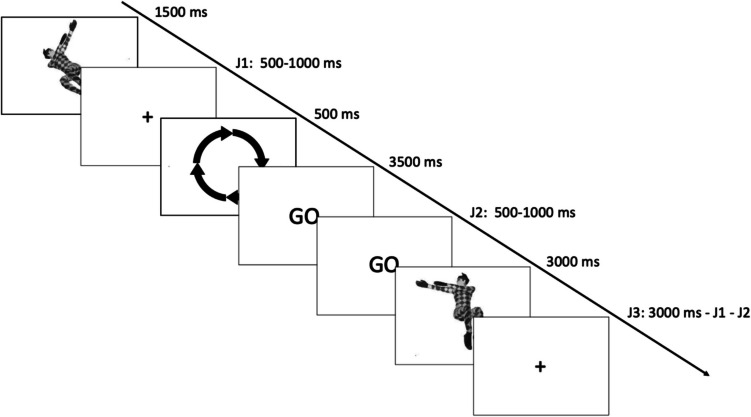
Example of experimental trial. Participants were asked to mentally rotate the first stimulus in the direction shown by the arrows to the vertical axis of the screen. Then, participants indicated whether the second stimulus (always vertical) represented the same or different item with respect to the first stimulus. In the example reported in the figure, the correct response is “different.”

Participants performed MR while behavioral and fMRI data were recorded. In each of the 6 runs of the experiment, participants were shown 60 pairs of stimuli (30 pairs per block) belonging to the same type of stimulus (abstract object, human body, real object). For each pair, the first stimulus showed an item rotated by one of ten rotation angles (± 30°, ± 60°, ± 90°, ± 120°, and ± 150°) with respect to vertical. This stimulus was presented for 1500 ms, tailed by a blank screen of a jittered duration (from 500 ms to 1,000 ms). Then, a cue appeared for 500 ms, representing a circular arrow turning in a clockwise or counterclockwise direction, followed by a screen displaying the word “GO” for 3,500 ms. During this time, participants were instructed to mentally rotate the first stimulus in the direction indicated by the arrow, so that the main axis of the stimulus would align with the vertical axis on the screen. This target orientation was defined purely spatially (i.e., aligned vertically), regardless of the item’s common orientation. For example, even real-world objects, such as bike brakes—typically seen with the long bar in a horizontal orientation—had to be mentally rotated towards the vertical axis of the screen. Participants indicated when they completed this task by pressing a key. This action provided their RT for each trial (pair of stimuli). After the participant’s response, the word “GO” was visible with a jittered duration ranging from 500 ms to 1,000 ms. Then, the second stimulus of the pair was shown for 2,500 ms, representing the same or different item oriented vertically. Participants then pressed a key to indicate whether the second stimulus corresponded to the result of their MR of the first stimulus, further indicating the accuracy for each trial (Fig. 2).

In 50% of the trials, the answer was"same", in 50% of the trials the answer was"different."To avoid memorization, the “different” trials were further divided into two categories: “Mirrored,” where the second stimulus was a mirror picture of the first one; “Wrong direction,” where the second stimulus was rotated in the opposite direction with respect to the direction indicated by the arrow, resulting in a mismatch with the version of the first stimulus mentally rotated by the participant. The three types of stimuli (abstract objects, human bodies, real objects) were presented in a randomized order. To familiarize the task, participants underwent a training session of four trials for each type of stimulus outside the fMRI scanner. The stimuli used in the training session were different with respect to those used in the experiment.

### MRI acquisition

We used a Siemens Magnetom Prisma Fit 3 T scanner and a 64-channel head coil (Siemens, Erlangen, Germany) to acquire both functional and anatomical brain images. A T2*-weighted echo-planar imaging sequence employing an isotropic voxel size of 2 mm^3^ (2 × 2 × 2 mm), echo time/repetition time (TE/TR) of 30/2,000 ms, 64 slices, simultaneous multislice factor of 2, field of view (FoV) of 192 mm^2^, flip angle of 80 degrees, matrix size of 96 × 96, interleaved ascending acquisition, and 2,136 Hz bandwidth per pixel was applied to get the functional brain images. The field of view was aligned parallel with the commissural line and included the whole cerebrum. Dummy scans were acquired to establish steady-state magnetizations prior to each functional acquisition. To account for spatial distortion in the functional brain images, we acquired a pair of spin echo images with opposite phase encoding directions, matching the orientation of the functional scans. These spin echo images were obtained at the beginning of each run, for a total of three sequences. All MR stimuli were presented using PsychoPy (psychophysics software in Python (Peirce et al., 2019) and delivered through MRI-compatible mirror attached to the head coil. For each of the six blocks (2 per experimental condition) of the MR task, we collected 171 functional image volumes (6 min 20 s), including 6 additional volumes for safety purposes. To synchronize code and scanner, the functional acquisition initiated the presentation code. Anatomical brain images were acquired using a T1-weighted magnetization prepared rapid gradient echo (MP2RAGE) sequence (Marques et al., 2010), isotropic voxel size = 1 mm^3^, FoV = 256 mm^2^, TE/TR_mprage_ = 2.9/5,000 ms, 192 contiguous sagittal slices, inversion time (TI1)/flip angle = 700 ms/4°, TI2/flip angle = 2500 ms/5°, matrix size = 240 × 240, duration = 3 min and 30 s.

We additionally recorded RT and accuracy during the runs. Data were stored in.txt files to be evaluated post-hoc.

### Data analysis

#### Behavioral data

We collected accuracy and RT data. Responses were filtered for errors and RT outliers above/below 3 standard deviations (SD) from the group mean specific for each type of stimulus. Statistical analyses were performed with RStudio (https://rstudio.com/), comprising repeated-measure analyses of variance of the mean RTs and accuracy, with gender as a fixed factor and stimulus as a random factor. Main effects and interactions were considered significant according to a level of significance of 0.05. If a significant effect was found, post-hoc analyses were performed using *t*-tests with the *p-*values adjusted for multiple comparisons according to the Bonferroni method.

#### MRI quality assessment process

MRI data were converted using the Dcm2Bids program (https://github.com/cbedetti/Dcm2Bids) from DICOM format to the Brain Imaging Data Structure (https://bids.neuroimaging.io/). The MRI Quality Control tool (Esteban et al., 2017) was then used to assess the quality of the structural and functional data. Using the MRIQCeption tool (https://github.com/elizabethbeard/mriqception), a comparison was done between the acquired quality metrics and a set of metrics from the MRI Quality Control tool online application program interface (Esteban et al., 2019).

#### MRI data preprocessing

The fMRI data were preprocessed using fMRIPrep version 1.5.1rc2 (Esteban et al., 2019) a based on Nipype (Gorgolewski et al., 2011). The T1-weighted (T1w) volumes underwent intensity non-uniformity correction using N4BiasFieldCorrection (Tustison et al., 2010) distributed with ANTs 2.3.3 (Avants et al., 2008) and used as T1-weighted-reference throughout the workflow. They were then skull-stripped using antsBrainExtraction.sh v2.2.0, based on the Open Access Series of Imaging Studies (OASIS) template. Brain surfaces were reconstructed using recon-all from FreeSurfer v6.0.1 (Dale et al., 1999), and the brain mask obtained previously was further refined with a customized approach reconciling ANTs-derived and FreeSurfer-derived segmentations of the cortical gray matter from Mindboggle (Klein et al., 2017). To achieve spatial normalization, data were registered to the International Consortium for Brain Mapping (ICBM) 152 Nonlinear Asymmetrical template version 2009c (Fonov et al., 2009) using the antsRegistration tool of ANTs v2.2.0 (Avants et al., 2008). This registration involved brain-extracted versions of both the T1w volume and the template. Subsequently, brain tissue segmentation for cerebrospinal fluid (CSF), white matter (WM), and gray matter (GM) was performed on the brain-extracted T1w data using fast (FSL v5.0.9; Zhang et al., 2001). The functional data underwent slice time correction using 3dTshift from AFNI v16.2.07 (Cox, 1996) and motion correction using mcflirt (FSL v5.0.9; Jenkinson et al., 2002). Distortion correction was performed using an implementation of the TOPUP technique (Andersson et al., 2003) with 3dQwarp (AFNI v16.2.07; Cox, 1996). Co-registration to the corresponding T1w data was done using boundary-based registration (Greve & Fischl, 2009) with six degrees of freedom, employing bbregister (FreeSurfer v6.0.1). The transformations for motion correction, field distortion correction warp, BOLD-to-T1w transformation, and T1w-to-template (MNI) warp were combined and applied in a single step using antsApplyTransforms (ANTs v2.2.0) with Lanczos interpolation.

To handle physiological noise, a Component Based Noise Correction Method (CompCor, Behzadi et al., 2007) was used to extract principal components for the anatomical CompCor variants (aCompCor). A mask excluding cortical signal was created by eroding the brain mask, leaving only subcortical structures. Six aCompCor components were calculated within the intersection of the subcortical mask and the union of CSF and WM masks derived from the T1w data, projected to the native space of each functional run. Frame-wise displacement (FD) and the Derivative of RMS variance over voxels (DVARS; Power et al., 2014) were calculated for each functional run using the Nipype implementations.

Further processing involved masking the functional data using the brain mask obtained from fMRIPrep. Fourteen fMRIPrep-derived confounds (six motion parameters, FD, standardized DVARS, and six aCompCor components) were removed at a voxel-wise level using the Denoiser tool (Tustison et al., 2010). Finally, the functional data were spatially smoothed using a Gaussian kernel with a full-width at half-maximum of 6 mm.

#### First-level GLM analysis

For the first-level General Linear Model (GLM) analysis, FSL FEAT was used (www.fmrib.ox.ac.uk/fsl). A separate GLM model was constructed for each participant and each run, where the three stimuli (abstract objects, human bodies, real objects) served as the regressors of interest, and their temporal derivatives were included as regressors of no interest. The regressors were convolved with a double-gamma hemodynamic response function and timed with the beginning and end of the video stimulus. We used FMRIB’s Improved Linear Model (FILM) prewhitening to adjust for autocorrelation, and a high-pass filter with a 100-s cutoff was used to remove low-frequency drifts.

#### Group-level GLM analysis

Using mixed effects (FLAME 1) as implemented in FSL, a whole-brain group-level analysis was performed to determine mean group effects. A cluster-based method was used to threshold the statistical map, with a threshold of *z* > 2.3. Family-wise error (FWE) correction was then applied to make adjustments at *p* = 0.05. The appropriateness of this approach in capturing the nuanced activations tackled by the present study was in line with and replicated previous work on MR (Bersier et al., 2025) and closely related topics, such as visuospatial reasoning (Gavazzi et al., 2022), spatial working memory (Ibrahim et al., 2017), and the development of spatial skills (Moen et al., 2020).

#### Univariate analysis

The purpose of the univariate analysis was to identify brain regions that showed significant activity during MR tasks, particularly in relation to stimulus type and performance levels. We sought to concentrate on the brain mechanisms directly linked to mental transformation by separating the 4-s interval after the cue. This analysis allowed us to explore how stimulus type modulates neural activity, contributing to our understanding of neural efficiency and compensatory mechanisms in MR.

Six separate contrasts were created to examine the distinctive neural activations corresponding to each stimulus type, with group added as a regressor to determine whether any observed effects were influenced by the gender using two-sample unpaired *t*-tests. To investigate potential links with accuracy, this covariate was added in a separate model. Accuracy was mean centered by subtracting the overall mean accuracy from each individual score and added as a third explanatory variable in the model, for both positive and negative effect. To investigate the relationship between neural activity and task performance, we compared the distributions of brain activity and accuracy. To this aim, we extracted the mean BOLD response intensity for each significant cluster identified in the group-level analysis, plotted it against the behavioral accuracy for each participant, and computed the Pearson correlation coefficients to assess the strength and direction of the relationship.

## Results

### Behavioral results

**Fig. 3:**
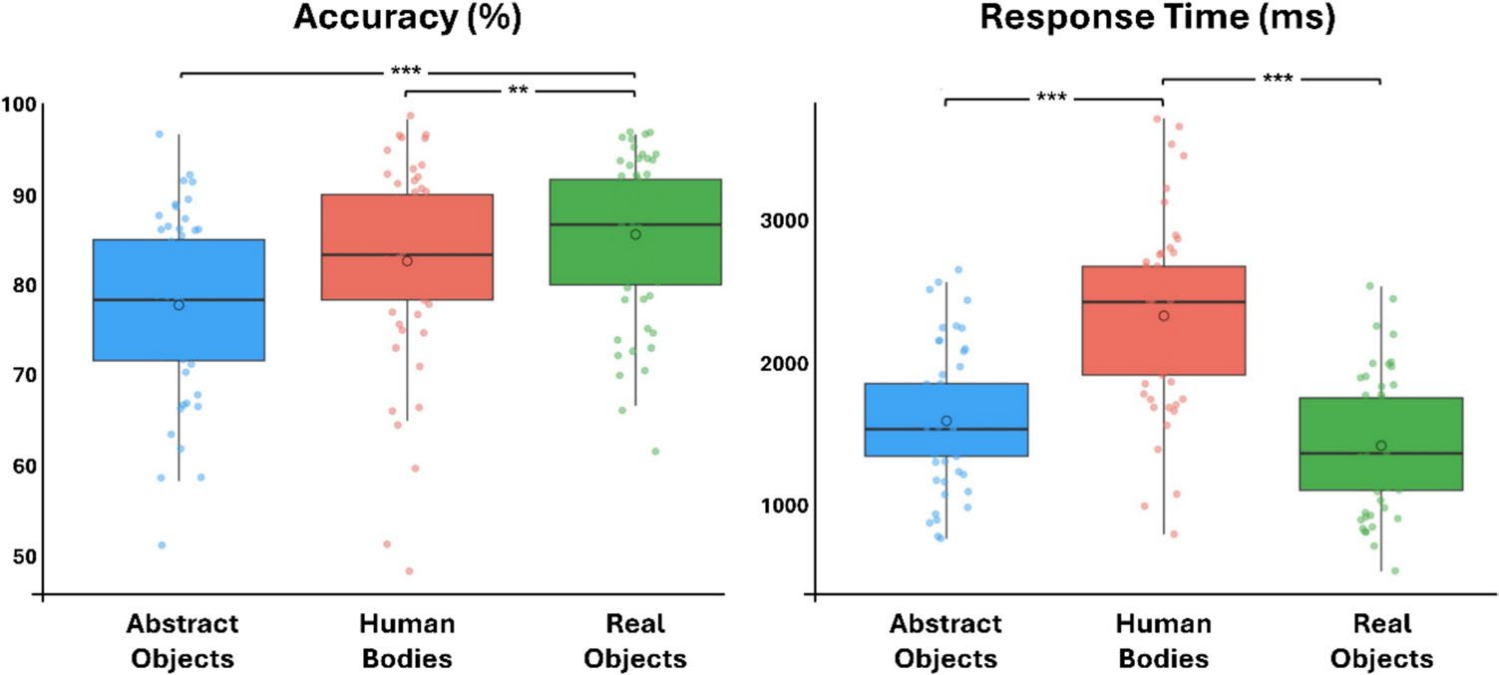
(Left) Accuracy: Bonferroni-corrected t-tests revealed that participants were significantly less accurate in MR of abstract objects with respect to human bodies and real objects. (Right) Response time: Bonferroni-corrected t-tests of the significant main effect of stimulus revealed that MR of human bodies was significantly slower with respect to abstract and real objects. (***p < 0.001; **p < 0.01)

The analysis of accuracy data revealed the significant main effects of gender [*F*(1,165) = 5.93, *p* = 0.015, *η*^2^ = 0.03] and stimulus [*F*(2, 165) = 10.10, *p* < 0.0001, *η*^2^ = 0.11]. The interaction between gender and stimulus was not significant [*F*(2,165) = 0.65, *p* = 0.052, *η*^2^ = 0.007]. The main effect of gender indicated that men had significantly higher scores (84.23%) than women (79.2%). Post-hoc analyses of the main effect of stimulus revealed that accuracy for the MR of real objects was significantly higher than MR of both abstract objects [*t*(1,110) =  − 4.65, *p* < 0.001, *d* =  − 0.87] and human bodies [*t*(1,111) =  − 3.61, *p* = 0.01, *d* =  − 0.49] (Fig. 3, left).

The analysis of RTs revealed the significant main effect of stimulus [*F*(2,165) = 50.02, *p* < 0.001, *η*^2^ = 0.37]. The main effect of gender [*F*(1,165) = 0.61, *p* = 0.43, *η*^2^ = 0.002] and the interaction between gender and stimulus [*F*(2,165) = 0.35, *p* = 0.7, *η*^2^ = 0.002] were not significant. Bonferroni-corrected post-hoc t-tests showed that MR of human bodies took longer (2,323 ms) than MR of abstract objects (1,588 ms) [*t*(2,103) =  − 7.25, *p* < 0.001, *d* =  − 1.35] and real objects (1,413 ms) [*t*(2,102) =  − 9.00, *p* < 0.001, *d* =  − 1.68] (Fig. 3, right).

### Neuroimaging results

The significant group contrasts for men and women during MR of abstract objects, human bodies, and real objects are summarized in Table 1.

**Table 1:** Significant clusters

	MNI			Hem	Peak Z	Cluster	
	x	y	z			Size	*p*
Abstract Objects; Men > Women							
Inferior frontal	48	38	12	Right	4.55	630	<.001
Middle frontal	38	32	16	Right	4.25		
Human Bodies; Men > Women							
Superior medial frontal	0	56	22	Right	3.62	434	0.011
Anterior cingulum	16	42	10	Right	3.27		
Superior frontal	26	62	4	Right	3.21		
Superior medial frontal	12	60	34	Right	3.38	387	0.02
Human Bodies vs. Abstract Objects; Women > Men							
Inferior parietal	40	− 50	42	Right	3.4	614	<.001
Superior parietal	26	− 68	58	Right	3.16		
Precuneus	− 2	− 74	58	Left	3.59	461	0.002
Precuneus	4	− 48	62	Right	3.26		
Superior parietal	− 20	− 74	56	Left	3.08		
Real Objects vs. Human Bodies; Women > Men							
Inferior occipital	26	− 102	−10	Right	3.8	412	0.003
Lingual gyrus	20	− 94	−12	Right	3.77		
fMRI-Accuracy Correlation; Real Objects; Men							
Precuneus	− 2	− 52	42	Left	3.55	304	0.04
Superior occipital	28	− 64	40	Right	3.23		
Precuneus	8	− 56	46	Right	2.97		
fMRI-Accuracy Correlation; Real Objects; Women							
Precentral	30	− 18	64	Right	3.89	432	0.01
Postcentral	28	− 26	62	Right	3.07		
Premotor	10	− 10	70	Right	3.52	362	0.03
Superior frontal	16	− 4	66	Right	3.47		
Premotor	− 6	− 4	60	Left	2.73		
Paracentral	− 4	− 14	72	Left	2.53		

#### Gender-specific brain activity

**Fig. 4:**
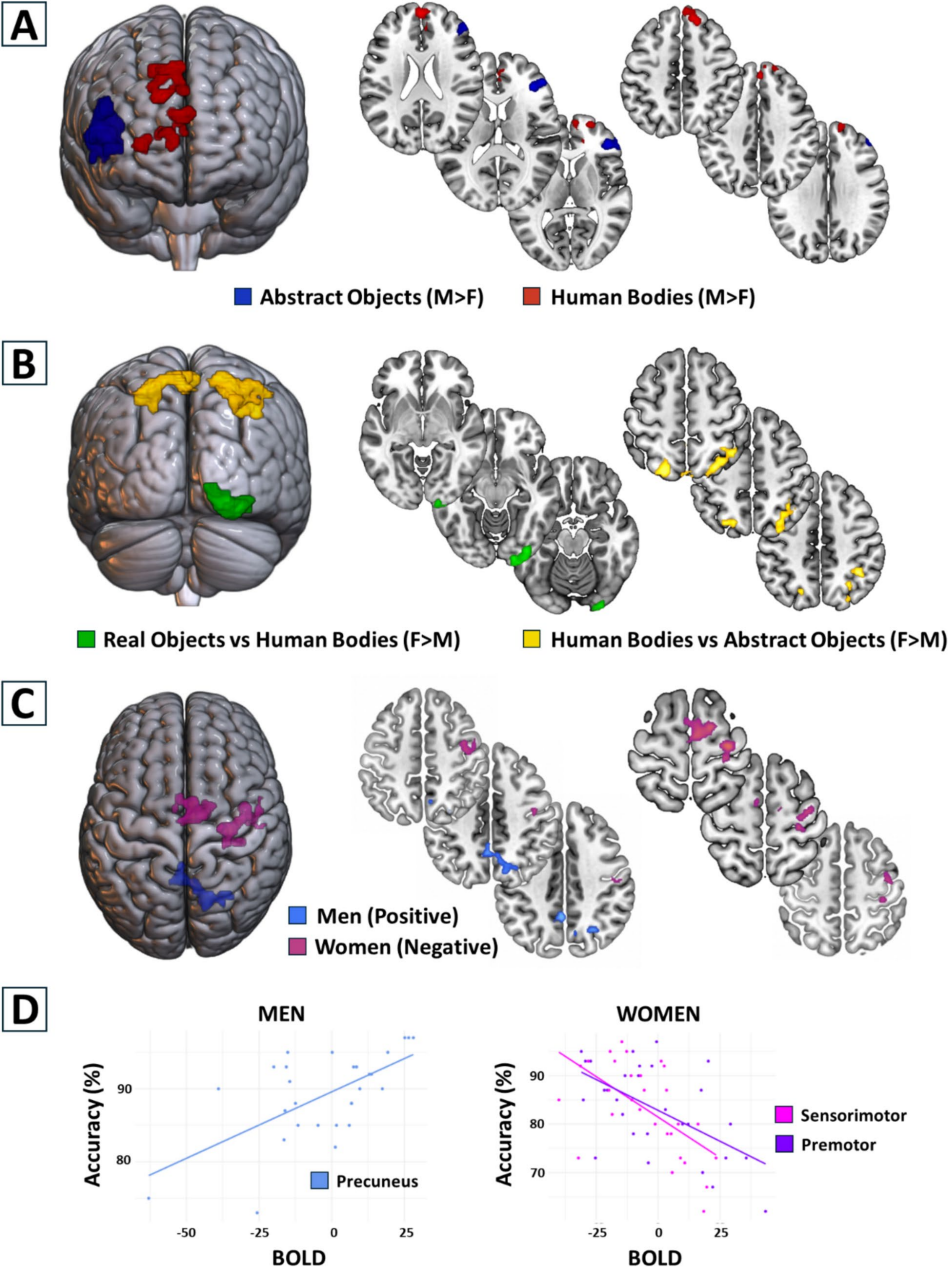
The results of whole-brain analysis are projected onto 3D brains (left column) and axial slices (right column). **(A)** Gender-specific brain activity associated with MR of different stimuli. The activity clusters represent the brain regions where men had larger activity compared with women for the MR of abstract objects (blue) and human bodies (red). **(B)** Brain activity-related relationships between gender and comparisons between stimuli. The activity clusters represent the brain regions where women had larger activity compared to men in the contrast between MR of real objects and human bodies (green), and between MR of human bodies and abstract objects (yellow). **(C)** Correlation between accuracy and brain activity during MR of real objects. The activity clusters represent the regions where accuracy and brain activity correlated, separated for men (blue) and women (magenta). **(D)** Scatterplots illustrating the correlation between accuracy and brain activity related to MR of real objects in men (left) and women (right). Accuracy and brain activity correlated positively in men (precuneus; left panel). In women there was a negative correlation between accuracy and brain activity in the sensorimotor (pink) and premotor clusters (purple) (right panel). All statistical maps shown in A, B, and C were computed with a cluster-based threshold of z > 2.3 FWE-corrected at *p* = 0.05

Taking into account stimuli-related differences, our analysis showed that men had larger activity during MR of abstract objects in a cluster covering the inferior and middle frontal lobe (peak coordinates = 48, 38, 12; peak *t*-value = 4.55; 630 voxels; Fig. 4A; Table 1). No significant clusters resulted for women compared to men during MR of abstract objects. For the MR of human bodies, we found that men had larger activity in two clusters covering the superior medial frontal lobe (Fig. 4A; Table 1). The larger and more posterior cluster comprised the superior medial frontal lobe and anterior cingulum (peak coordinates = 0, 56, 22; peak *t*-value = 3.62; 434 voxels). The smaller and more anterior cluster corresponded to the superior medial frontal lobe only (peak coordinates = 12, 60, 34; peak *t*-value = 3.38, 387 voxels in the cluster). No significant clusters resulted for women compared to men during MR of human bodies. No gender differences were detected in the brain activity associated with MR of real objects.

#### Gender-stimulus interactions

Contrasting the brain activity related to MR of human bodies against abstract objects, we found that women had larger activity in the parietal cortex (peak coordinates = 40, − 50, 42; peak *t*-value = 3.4; 614 voxels) and precuneus (peak coordinates =  − 2, 74, 58; peak *t*-value = 3.59; 461 voxels) (Fig. 4B; Table 1). The reverse contrast did not reveal any significant cluster. Contrasting the brain activity associated with MR of real objects versus human bodies, women showed larger activity in the occipital cortex (peak coordinates = 26, − 102, − 10; peak *t*-value = 3.8; 412 voxels) (Fig. 4B; Table 1). The reverse contrast did not show any significant cluster.

#### Gender-accuracy correlations

When accuracy was added as a regressor in the third level analysis, differences emerged for both women and men in the MR of real objects. For men, we found a positive correlation between MR accuracy and the activity of a cluster centered on the precuneus and comprising the superior occipital cortex (peak coordinates =  − 2, − 52, 42; peak *t*-value = 3.55; 304 voxels). For women, we found a negative correlation between MR accuracy and the activity of two clusters. The larger and more posterior cluster comprised the primary motor (precentral) and sensory (postcentral) cortices (sensorimotor cluster; peak coordinates = 30, − 18, 64; peak *t*-value = 3.89; 432 voxels). The smaller and more anterior cluster comprised the premotor cortex (peak coordinates = 10, − 10, 70; peak *t*-value = 3.52; 362 voxels) (Fig. 4C; Table 1). Scatterplots for each gender and cluster were generated using participant-specific BOLD response values extracted from the significant clusters in the group-level analysis. For women, we observed significant negative correlations between accuracy and brain activity in both the sensorimotor (*r* =  − 0.59, *p* = 0.001) and premotor cluster (*r* =  − 0.57, *p* < 0.001). For men, we found a significant positive correlation between accuracy and brain activity in the precuneus (*r* = 0.62, *p* < 0.001) (Fig. 4D).

## Discussion

The present study supports that gender modulates the brain activity associated with MR of different types of stimuli. Men and women differed in terms of both behavioral performance and brain activity, further depending on the type of stimulus. These findings suggest that gender differences in MR depend on the nature of the stimulus. While previous work focused mostly on the effects of binary distinctions in MR, the present study aimed to investigate the impact of a broader range of stimuli and belonging to different categories. We used MR of abstract objects, human bodies, and real objects to study whether nuanced differences in the nature of the stimulus would modulate the gender-specific behavioral and/or neural response to MR. With this experimental approach we observed that brain activity reflected stimulus-dependent gender differences, further shaped by the extent of stimuli’s embodiment and real-world familiarity.

### Men-specific brain activity related to MR

During MR of abstract objects, we found that men had larger activity in the inferior and middle frontal gyri compared with women. This finding is in line with previous evidence that men show larger inferior frontal activations despite equivalent cognitive performance (Bell et al., 2006). The apparent controversy between studies showing that the MR-related activity of the inferior frontal gyrus is larger in women than men (Hugdahl et al., 2006; Thomsen et al., 2000) or in men than women (Semrud-Clikeman et al., 2012) might be due to the small sample size (5 women and 6 men) of both these previous studies. Our results would confirm that the activity of inferior frontal gyrus is larger in men than women, further suggesting that this pattern is specific for abstract objects, because it was absent in MR of real objects and human bodies. Evidence that the use of a visuo-spatial strategy during MR is associated with inferior frontal activity (Bersier et al., 2024), reinforces our idea that men are more prone to use a visuospatial strategy, at least when it involves abstract objects or human bodies. Considering that (i) inferior frontal activity is associated with working memory (Baldo & Dronkers, 2006; Minamoto et al., 2010; Nixon et al., 2004; Rypma et al., 1999) and (ii) working memory plays a central role in MR (Hyun & Luck, 2007; Lehmann et al., 2014; Pardo-Vazquez & Fernandez-Rey, 2012; Wang et al., 2018), we propose that the differential inferior frontal activity in men and women may reflect a different effort in working memory to perform MR of abstract objects, which would be larger in men than women. This interpretation would be corroborated by the gender difference in the inferior frontal MR-related activity associated with anatomical brain differences (Blanton et al., 2004; Hammers et al., 2007; Harasty et al., 1997; Tomasi & Volkow, 2012). Alternatively, it is possible that gender differences in inferior frontal activity are driven by the inhibitory mechanisms required to retain responding before MR is completed. In fact, men can show greater inferior frontal activity during successful inhibition, while women can show greater engagement during unsuccessful inhibition (Weafer, 2020).

For MR of human bodies, our analysis indicated that men had larger activity in a portion of the frontal cortex corresponding to the medial prefrontal cortex. Within the frontoparietal network involved in MR (Cohen et al., 1996; Moen et al., 2020; Schendan & Stern, 2007), the prefrontal cortex is implied in monitoring aspects of the working memory required to perform MR (Petrides et al., 1993), is active while solving cognitive tasks related to MR (Çakır et al., 2011; Mutlu et al., 2020), and is important for MR-related visuo-spatial transformations (Johnston et al., 2004; Vingerhoets et al., 2001). In addition, the medial prefrontal cortex plays a central role in self-referential thought (D’Argembeau et al., 2007; Gusnard et al., 2001; Mitchel et al., 2005) and, in particular, in Theory of Mind (Frith et al., 2003; Lev-Ran et al., 2012), the ability to understand and attribute mental states to oneself and others. Notably, Theory of Mind correlates with MR (Lehmann & Jansen, 2019), predicts individual spatial abilities including MR (Viana et al., 2016) and, together with MR, is part of mental spatial transformation abilities (Frick et al., 2014). Evidence that transcranial electrical stimulation of the medial prefrontal cortex improves Theory of Mind in women but not in men (Adenzato et al., 2017) suggests that in men it is already strongly involved in Theory of Mind. On this basis, we propose that the larger activity in the medial prefrontal cortex of men may reflect a stronger reliance on self-related Theory-of-Mind-like reference frame in parallel with a stronger cognitive effort to perform MR. This mechanism would be further modulated by the manipulability of the MR stimulus, in that it would come into force for abstract objects and human bodies, which are less “manipulable” with respect to real objects. This would bring that, with respect women, men would more strongly rely on self-referred cognitive processing even if MR concerns relatively less manipulable objects.

### Women-specific brain activity related to MR

Contrasting MR of human bodies against abstract objects, our analysis indicated that women had larger activity in superior and inferior parietal cortices compared to men (Table 1; Fig. 4B). This result hints to previous findings that the superior parietal cortex is (i) more active during egocentric than allocentric MR (Tomasino & Gremese, 2016) and (ii) is particularly relevant for MR of objects (Harris & Miniussi, 2003). The larger activity that we found in women could reflect that women tend to rely on an egocentric strategy more than men. This interpretation is in line with previous findings that women are more likely than men to use egocentric cognitive processing to solve MR tasks (Bersier et al., 2024), tend to employ egocentric perspectives in MR of human-like stimuli (Muto, 2021), and show strong brain activity for egocentric MR of bodily stimuli (Seurinck et al., 2004).

In apparent contrast with previous evidence that occipital regions are recruited during MR of full body stimuli (Blanke et al., 2010; Perruchoud et al., 2016; Zacks, 2008), we found that women had larger occipital activity than men during MR of real objects than human bodies. However, because previous studies merged men and women in one sample, it could be possible that the obtained results were driven by eventual effects brought by only one part of the sample. Based on the observation that in women the occipital regions were more active during MR of real objects than human bodies, our findings suggest that the localization of brain activity related to MR depends on gender. This finding indicates that, as a function of gender, the activity of regions traditionally linked to MR of human bodies (the occipital cortex), can in fact encode MR of a different stimulus (real objects). Because the activity in this region reflects visuospatial processing, we propose that women prefer visuospatial strategies to perform MR of stimuli (real objects) that otherwise would recruit different strategies (likely sensorimotor) due to possible overpowering by men’s brain activity in previous studies.

### Correlation between MR accuracy and brain activity

We observed that the correlation between accuracy and brain activity was significant only for MR of real objects, with opposite effects between men and women. In men, increased accuracy was related to increased activity in the precuneus. This region is typically affiliated with MR (Barriga, 2015), and the use of an object-based visuospatial strategy to solve MR tasks (Boccia et al., 2014; Lambrey et al., 2012; Zhang & Ekstrom, 2013). This strategy is often proposed to be more effective, because it is faster and less error-prone, compared to an effector-based strategy (Halpern, 2013; Linn & Petersen, 1985; Shepard & Metzler, 1971). Some studies have hyped that men's superior performance in MR tasks could be explained by a spontaneous adoption of this strategy. For instance, Butler & colleagues (2006) found that MR of abstract objects is associated with higher accuracy and larger activation of the precuneus in men than women. The precuneus is involved in viewpoint and array rotation tasks too (Lambrey et al., 2012), where men have larger activity in the precuneus and right inferior frontal gyrus than women in spatial perspective tasks (Kaiser et al., 2008). On this basis, we propose that the correlation between accuracy and activity in the precuneus for the MR of real objects in men represents the employment of a visuospatial strategy to solve MR, which would not be the preferred strategy in women. Thus, the present finding suggests the existence of gender-specific neuro-behavioral patterns bound to the cognitive strategy adopted in MR tasks.

For women, we found a negative correlation between MR accuracy and the activity of both the sensorimotor and premotor clusters: The larger the brain activity, the lower the accuracy. Sensorimotor regions are typically associated with the employment of motor strategies and an egocentric frame of reference to solve MR tasks (Bersier et al., 2024; Kosslyn et al., 1998; Vingerhoets et al., 2002). Our results indicate that women would rely on motor strategies for MR of real objects. The reason for this choice can be explained in the light of the characteristics of this stimulus. Real objects have a strong affordance of being graspable, and we selected them with the specific criterium of being manipulable with only one hand. In this vein, Grèzes et al. (2003) showed that intrinsic properties of an object can affect the motor responses consistent or not with the action typically associated with the object itself. In particular, they found that motor responses were faster for objects that allowed a consistent than inconsistent grip, showing that graspable objects are treated differently at the cognitive level. For MR however, it seems that exploiting the motor properties of manipulable objects is not the best way to proceed. Our results support this idea, in that visually presented real objects would automatically initiate some aspects of action simulation, especially in women. Moreover, Long et al. (2021) highlighted that gender differences in MR are mediated by the functional connectivity between the default mode network and the salience network. Men may rely more on visuospatial networks linked to the default mode network and the salience network, while women may engage more motor-related processes, as indicated by the differences in accuracy and brain activity when rotating real objects in our study. In our case, we observed a negative correlation between accuracy and the sensorimotor cluster in women during MR tasks involving real objects. This finding suggests that while motor areas are automatically activated, relying on motor strategies in these tasks may in fact interfere with the efficiency of a visuospatial strategy, leading to decreased accuracy in women. This supports the hypothesis that action simulation triggered by graspable objects, while useful in some contexts, may not always be the most effective strategy for MR tasks, particularly in women.

In sum, the positive relationship between accuracy and brain activity in the precuneus in men indicates that higher accuracy is associated with more efficient neural processing in this region. Conversely, women showing a negative relationship between accuracy and brain activity in the sensorimotor areas suggests that increased brain activity in these regions may not translate into higher accuracy, potentially indicating less efficient neural processing. Further research could explore these differences in neural efficiency, examining how they impact cognitive performance and whether training can enhance efficiency in specific brain regions.

### Behavioral effects

While we predicted that gender would have influenced both RT and accuracy, we found that only accuracy was significantly different between genders: higher in men, lower in women. Although small (*η*^2^ = 0.03), this effect confirms previous evidence that men have an advantage in visuospatial tasks (Halpern, 2013; Linn & Petersen, 1985; Shepard & Metzler, 1971; Voyer et al., 1995; Zapf et al., 2015). This dissociation between RT and accuracy confirms previous findings (Boone & Hegarty, 2015, 2017; Quaiser-Pohl et al., 2014) and may reflect differences in measurement sensitivity. One possible explanation is that, in our setup, RT is a coarser or more “gross” measure compared to accuracy, in that RTs were measured on the basis of introspective evaluations made by the participants themselves about when they completed each MR trial. Conversely, being independent from individual ability to precisely analyze the precise timing of a cognitive process, accuracy may be a more fine-grained measure, at least in our setup. This difference would make accuracy more sensitive to subtle differences related to gender or our experimental manipulations. Thus, the absence of an RT effect may not reflect the absence of underlying differences, but rather the limits of what this measure can detect in our experimental approach.

In addition, we found that MR of different stimuli resulted in different accuracy and RT (Fig. 3). Participants showed higher accuracy in MR of real objects compared to human bodies and abstract objects. This result is in line with previous evidence that mental spatial transformations of real objects are easier than abstract objects (Iachini et al., 2019), visual complexity of MR stimuli affects performance Jordan et al. (2002), and MR accuracy is better for visually more familiar than less familiar items (Doyle & Voyer, 2018; Koriat & Norman, 1985; Ruthsatz et al., 2014). On this basis, we propose that our participants were more accurate in MR of real objects because this type of stimuli seems more concrete, less visually complex, and more familiar compared to human bodies and abstract objects.

Finally, the significant main effect of stimulus on RTs showed that MR of human bodies was significantly slower than abstract and real objects. This finding confirms previous evidence that human bodies might involve more complex cognitive processing, possibly due to the differences between object-based visuospatial reasoning (objects) and body-centered embodiment (human bodies; Amorim et al., 2006; Doyle et al., 2016; Jansen et al., 2020; Krüger et al., 2014). Zacks (2008) suggested that in MR of bodily stimuli could trigger embodiment processes that may increase cognitive load and result in slower RTs. Conversely, when MR is framed as a purely spatial manipulation, participants may rely more on visuospatial strategies, leading to faster RTs. Thus, the differences in how the MR task is presented may account for the variability in findings across stimuli, suggesting that embodiment processes might be contingent on how participants approach MR. On this basis, we suggest that the longer RTs for MR of human bodies indicated that embodiment was at play, albeit not resulting in higher accuracy, thus corroborating results from Krüger & colleagues (2014).

### Limitations

First, it would be worth noting that our fMRI data analysis used a whole-brain approach. This approach may have inherent limitations in that, for instance, conducting numerous statistical tests across the whole brain may increase the risk of false positives. To minimize this risk, and in line with previous work, we combined this approach with statistical correction methods for multiple comparisons, employing a strict and commonly accepted thresholding procedure. While, in principle, the whole-brain approach may have inherent limitations, we note that it offers a comprehensive view of brain activity, capturing eventually unexpected activations, especially in relatively exploring studies like the present one. Building on the initial findings provided by holistic whole-brain analyses, it is possible to formulate new and more specific hypotheses. In this framework the present study may constitute a reference for future studies about more specific aspects of the interaction between stimulus and gender in MR. These studies may include, for example, 1) investigating the cognitive strategies employed by men and women during MR tasks to provide insights into the differential brain activity, and 2) developing gender-specific training programs to enhance MR performance and leverage distinct brain activity patterns.

Second, we predominantly interpret the results through the lens of gender differences in cognitive strategies, positing that men and women employ distinct visuospatial or motor-based approaches contingent upon the type of stimulus. Although plausible, this interpretation remains relatively speculative, since other sources of individual variability may also contribute to the observed effects. Additional sources of variability may include spatial training, cognitive style, and developmental history. For instance, we cannot exclude that the samples of men and women differed in terms of spatial training, which can influence the accuracy and RTs of MR (Meneghetti et al., 2016), or cognitive style, which can play a key role in both behavioral and neural aspects of MR (Bersier et al., 2025). Accordingly, we recently reported that neuro-behavioral aspects of MR are influenced by the interaction (not isolation) between gender and cognitive strategy (Bersier et al., 2024). In addition, MR can be influenced by many other factors, including sex hormones (Bernal & Paolieri, 2022; Bernal et al., 2020; Gurvich et al., 2023; Hausmann et al., 2000; Scheuringer & Pletzer, 2017), nicotine (Neumann et al., 2007), caffeine (Smith et al., 1990), sleep (Debarnot et al., 2013), and circadian chronotype (Nishida et al., 2022). Altogether, while we acknowledge that attributing the present study’s findings solely to gender risks oversimplifying a complex phenomenon, we also note that the attention deserved by the many possible sources of variability in MR should be the focus of future studies.

Third, while our study focused the differences between biologically established gender, growing evidence suggests that the individually perceived gender may influence MR. Folkierska-Żukowska et al. (2020) reported that gender-nonconforming homosexual men show distinct MR-related brain activity in the precuneus and superior frontal gyrus, regions that we also identified. Similarly, Rahman and Wilson (2003) found that heterosexual men outperformed homosexual men in spatial tasks, suggesting differences in parietal functioning. Xu et al. (2020) further demonstrated that homosexual men typically perform between heterosexual men and women on MR tasks, consistent with a cross-sex shift model. Taken together, these findings highlight the importance of considering individual differences beyond gender and suggest that MR may be influenced by a combination of biological, developmental, and identity-related factors.

## Conclusions

Our study supports a nuanced influence of the interaction between gender and stimulus type on the brain activity related to MR. In men we found larger prefrontal activity during MR of abstract objects and human bodies, suggesting a stronger reliance on self-referential cognitive processing. Additionally, in men we observed increased activity in the inferior and middle frontal gyri, indicating a greater effort in working memory and visuospatial strategies during MR. Conversely, in women we obtained larger activity in the parietal cortex, reflecting a preference for egocentric cognitive processing across different stimuli. Furthermore, in women we noted larger occipital activity during MR of real objects, suggesting a gender-dependent localization of brain activity. Notably, MR accuracy correlated with brain activity differently for men and women: men had increased accuracy with larger activity in the precuneus, while women had decreased accuracy with larger activity in sensorimotor regions. These findings highlight the intricate interplay between gender, cognitive strategies, and brain activity patterns, providing valuable insights into the neural mechanisms underlying MR and emphasizing the complex and nuanced ways in which gender and stimulus type interact to influence visuospatial reasoning.

## Data Availability

Deidentified data used in this research are publicly available on the following GitHub repository: https://github.com/nadiaBRS/Stimulus-specific-influence-of-gender-on-mental-rotation-related-brain-activity.

## Code availability

Analysis code used in this research are publicly available on the following GitHub repository: https://github.com/nadiaBRS/Stimulus-specific-influence-of-gender-on-mental-rotation-related-brain-activity.
